# Association of Epstein Barr Virus Infection (EBV) with Breast Cancer in Rural Indian Women

**DOI:** 10.1371/journal.pone.0008180

**Published:** 2009-12-04

**Authors:** Deepti Joshi, Munira Quadri, Neha Gangane, Rajnish Joshi, Nitin Gangane

**Affiliations:** 1 Department of Pathology, Mahatma Gandhi Institute of Medical Sciences, Sevagram, Maharashtra, India; 2 Mahatma Gandhi Institute of Medical Sciences, Sevagram, Maharashtra, India; 3 Department of Medicine, Mahatma Gandhi Institute of Medical Sciences, Sevagram, Maharashtra, India; George Washington University, United States of America

## Abstract

**Introduction:**

Breast cancer is the most common malignancy affecting females worldwide but conventional risk factors are able to explain only a small proportion of these cases. A possible viral etiology for breast cancer has been proposed and Epstein-Barr Virus (EBV) is a widely researched candidate virus. The aim of the present study, first one of its kind from India, was to determine if there is a greater association of EBV infection with breast cancer patients as compared to patients with benign breast diseases.

**Methods:**

We looked for expression of Epstein-Barr Virus Nuclear Antigen-1 (EBNA-1) in breast cancer tissue specimens by employing immunohistochemistry (IHC). We also measured levels of anti-EBNA-1 Immunoglobulin (IgG) antibodies in stored sera of these patients using commercial Enzyme linked Immunosorbent Assay (ELISA) kit. Patients with benign breast diseases were used as a comparison group for both immunohistochemical and serological analysis.

**Results:**

58 cases of malignant breast disease and 63 of benign breast disease (controls) were included in the study. Using manufacturer determined cut-off of 3 IU/ml, 50/55 tested (90.9%) cases and 27/33 tested (81.8%) controls were seropositive for anti-EBNA-1 IgG. Mean antibody levels were significantly higher for cases (54.22 IU/ml) as compared to controls (18.68 IU/ml). IHC for EBNA-1 was positive in 28/51 cases (54.9%). No IHC positivity was noted in the tested 30 controls. Our results show that EBNA-1 expression is seen in a significant proportion of breast cancer tissue specimens from rural India and as compared to patients with benign breast diseases these patients also have a higher immunological response against EBNA-1.

## Introduction

Breast cancer is the most common malignancy affecting women world-wide with an annual incidence of around 800,000 cases.[1], [2] Though various reproductive and hormonal factors have been identified as risk factors for breast cancer, but even together these factors do not explain more than fifty percent of all cases of breast cancer.[1], [3] This prompted the researchers to look for other risk factors including a possible viral etiology for breast cancer. Mouse mammary tumor virus (MMTV) has been implicated in causing mammary carcinoma in mice but search for similar viral sequences in humans have not yielded conclusive results.[4] Since then many other candidate viruses like Epstein-Barr Virus (EBV) and Human Papilloma Virus (HPV) have been proposed.[5], [6]

A possible association of EBV with breast cancer was thought of because of the following observations: High incidence of male breast cancers has been reported in Mediterranean countries which are endemic for EBV, some EBV associated lymphomas are known to occur in breast and there are morphological similarities between medullary carcinoma of breast and nasopharngeal carcinoma (NPC), an EBV associated malignancy.[4] EBV has also been found in breast tissue and milk, lymphoblastoid cell lines bearing EBV can infect mammary epithelial cells in vitro and transfection of p31 fragment of EBV DNA immortalizes epithelial cells including mammary epithelial cells.[5] If a causal association of EBV with breast cancer is established, it would have therapeutic significance because of the possible role of EBV specific cytotoxic T cells in targeting EBV associated tumor cells.[7]

Most of the studies detecting association of carcinoma breast with EBV infection have been carried out in western countries, but in-order to establish causation consistent results have to be demonstrated from studies conducted all over the world [8], [9] as EBV infection is prevalent worldwide. So far only few such studies have been reported from Asian countries and none from India. The aim of the current study was to determine if there is a greater association of EBV infection in female breast cancer patients from rural India as compared to those with a benign breast disease. For investigating this association we performed immunohistochemistry (IHC) for Epstein Barr Virus Nuclear Antigen-1 (EBNA-1) in breast tissue specimens, and also looked for presence of antibodies to EBNA-1 in serum samples of patients with benign and malignant breast diseases. Of the various known antigens of EBV expression, EBNA-1is essential for maintenance of viral episome and is expressed in all known forms of viral latency. [10] To the best of our knowledge this is the first study in which both serology and IHC for presence of viral protein has been done on the same study subjects.

## Methods

### Ethics Statement

This study was approved by the Mahatma Gandhi Institute of Medical Sciences (MGIMS), Institutional review board for research in human subjects located at Sevagram, India (Registered with United States Department of Health and Human services, registration #IRB00003623). Patients who were at least 18 years of age, did not have a relative with breast cancer were approached for participation in the study. Those who provided a written informed consent were included.

### Setting

This prospective case-control study was conducted at MGIMS, Sevagram, a rural based teaching hospital in central India, to determine the association between EBV infection and breast cancer. MGIMS is part of a nation-wide cancer registry, and in 2001–04 about one-fifth of all malignancies in women were breast neoplasms. Breast cancer is second only to cervical cancer in rural population based cancer registry in India, with age adjusted rate of breast cancer estimated to be about 9.7 per 100,000 population.[11]

### Cases and Controls

All consecutive women who underwent surgical excision for diagnosis or treatment of their breast lumps were considered for inclusion in this study. A study investigator approached the patients in the surgical ward, within 48 hrs of excision biopsy or mastectomy. Patients were informed about the subsequent study procedures and informed consent was sought. Patients who were at least 18 years of age, did not have a relative with breast cancer and provided written informed consent were included in the study. A detailed clinical and reproductive history was obtained from all such patients, a serum sample was collected and all serum samples were stored at −70 degree centigrade at this time.

The breast tissues from these patients were received in the histopathology services, grossed and subjected to tissue processing. Based on histopathological examination, the included patients were classified as cases if there was presence of invasive breast cancer or carcinoma-in-situ. Women whose breast lesions had either of the following diagnoses: Fibroadenosis, Duct ectasia, Fibroadenoma, Fibrosis, Mastitis, or mild hyperplasia without atypia, ordinary cysts (gross or microscopic), simple apocrine metaplasia (no associated hyperplasia or adenosis), or squamous metaplasia were classified as controls. Patients with lesions such as sclerosing adenosis, fibraoadenoma with complex features, moderate or florid hyperplasia without atypia, or atypical ductal or lobular hyperplasia were excluded from being either a case or a control. All histopathological slides were re-analysed independently by two trained pathologists and a consensus diagnosis was obtained. In cases of malignancy, size of the tumor, histological type of the tumor, grade of the tumor, and presence of metastatic lymphnodes were also noted.

### Immunohistochemistry(IHC)

We used paraffin embedded sections taken on amino-propyl-triethoxysilane coated slides for IHC. Tissue was fixed in 10% formalin. All paraffin blocks were stored at room temperature before further processing. The paraffin sections were deparaffinized and pretreated in 10 Mm citrate buffer (ph 6) in microwave oven for 20 minutes. Endogenous peroxidase activity was blocked by 3% hydrogen peroxide. After washing with phosphate buffered saline, slides were treated with power block reagent (BioGenex, San Ramon, CA, USA) and then with primary monoclonal antibody clone 0211 (AbD Serotec, Raliegh, NC, USA) at a dilution of 1∶10 for one hour. Bound antibody was detected using polymer horse radish peroxidase(HRP) conjugated anti-rabbit and anti- mouse antibody. (BioGenex, San Ramon, CA, USA). Peroxidase activity was detected by using 3,3′ diaminobenzidine (DAB) as chromogen with hydrogen peroxide as substrate. Sections were then counterstained by hematoxylin. Known case of NPC was taken as positive control, addition of monoclonal antibody was omitted in negative control. All IHC tests were performed by the same trained laboratory personnel.

### Serology

ELISA for IgG antibodies against EBNA-1 was performed on serum samples of cases and controls using a commercial ELISA kit (Serion Immunodiagnostics, Wurzburg, Germany). The test was carried out using manufacturer's instructions. Briefly, 100 µL of standard diluted sera was pipetted into microtest wells and incubated for 60 minutes at 37°C. After subsequent washing, conjugate solution was added and incubated for 30 minutes at 37°C. After washing steps, addition of substrate solution and a second incubation (30 minutes at 37°C), reaction was stopped and extinction values were read at 405 nm. Continuous determination of antibody activity was done by using the provided standard curve. The test kit has inbuilt positive and negative controls, and their values need to be in specified range for quality control to be met. The assay run in this study met the quality control. The kit cannot reliably measure IgG antibody levels above 110 IU/mL, hence all high values are truncated at this point. Due to limited resources we performed serology in cases and half of randomly selected controls. Serology assay for all samples was performed by one supervised laboratory technician in a single sitting.

### Statistical Analysis

We performed a descriptive statistical analysis about distribution of key demographic, reproductive, IHC and serologic features between cases and controls. In addition we also compared tumor grade, and stage between those who were positive or negative for EBNA-1 expression on IHC. The difference between continuous and discrete variables was compared using student's t-test and chi square tests respectively and p<0.01 was used to classify difference between two groups as significant. We also compared the level of anti- EBNA-1 antibodies in serum samples between cases and controls as a continuous measure. All statistical analysis was done using statistical software STATA version 9.0.

## Results

**Figure 1:**
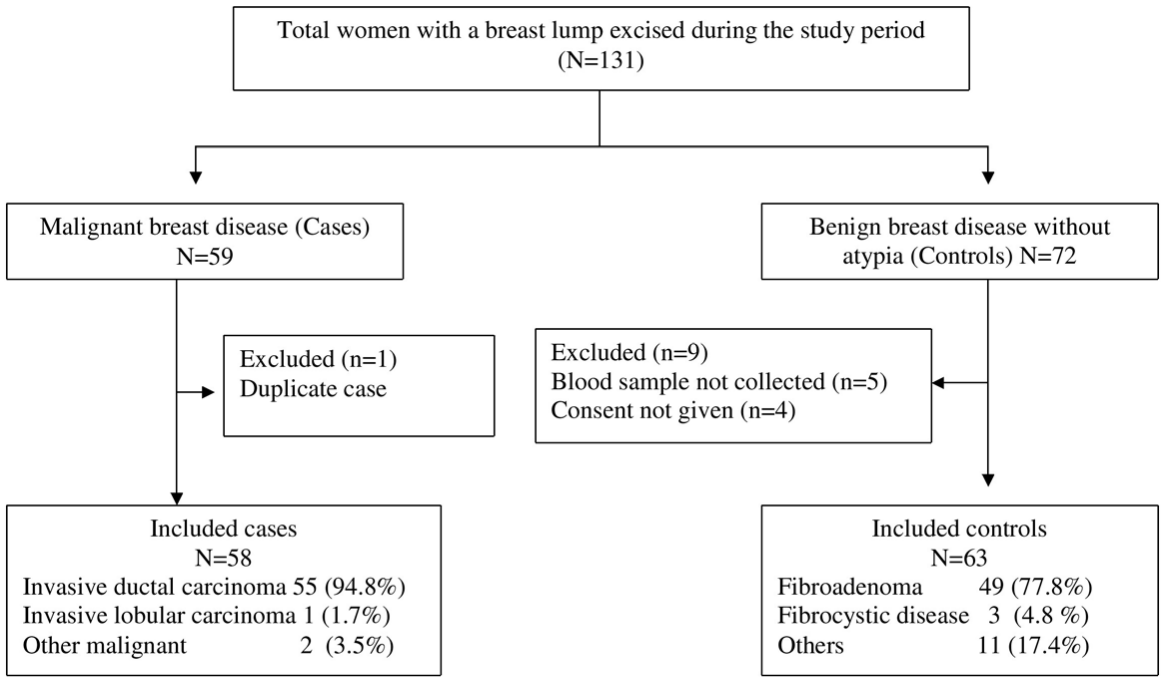
Study flow.

The study included a total of 131 patients who presented between January 2007 and March 2008. Of these patients a total of 121 were finally included in the study, 58 patients were classified as cases (malignant breast disease), and remaining 63 as controls.(Figure 1). The mean age of cases was 47.2 (S.D 10.2) years, and was significantly higher as compared to that of controls 27.5 (S.D 12.0) years (p<0.01). Due to this age difference, cases were also more likely to be married, had more pregnancies and as compared to controls, a higher proportion of women had lactated in the past. (Table 1)

**Table 1:** Baseline characteristics between those with benign and malignant lesions.

Variable	Benign (n = 63)	Malignant (n = 58)	P value
Age (years)	27.53 (12)	47.22 (10.2)	<0.01
Duration of lump (months)	14.86(18.8)	8.2 (9.5)	0.01
Respiratory infection past 6 months*	7 (11.1%)	5 (8.6%)	0.64
Age of menarche (years)	13.79 (1.98)	13.87 (1.18)	0.77
Years since marriage	10.95 (10.14)	18.20 (4.45)	<0.01
One or more conception (number)	29	57	
Age (years) at first conception†	20.58 (2.95)	20.56 (3.70)	0.97
Number of pregnancies†	2.33 (0.96)	3.08 (1.56)	0.02
Number of children†	2.22 (0.93)	2.80 (1.34)	0.04
Ever lactated*	28 (44.4%)	53 (91.3%)	<0.01
Oral contraceptive use*	4 (6.35%)	1 (1.72%)	0.202
Hormonal agents used *	2 (3.2%)	2 (3.4%)	0.93
EBNA-1 IgG serology done (number)	33	55	–
EBNA-1 IgG levels (IU/ml) Mean	18.68 (14.80)	54.22 (46.70)	<0.01
EBNA-1 IgG levels (IU/ml) Median	16	34	
EBNA-1 IgG levels (IU/ml) Range	0–50	0–110	
IHC for EBNA-1 done	30	51	<0.01
Positive (%)	0 (0)	28 (54.9)	

All values indicate mean (standard deviation) unless stated otherwise.

* Number (percentage).

† In those with one or more conceptions.

**Figure 2:**
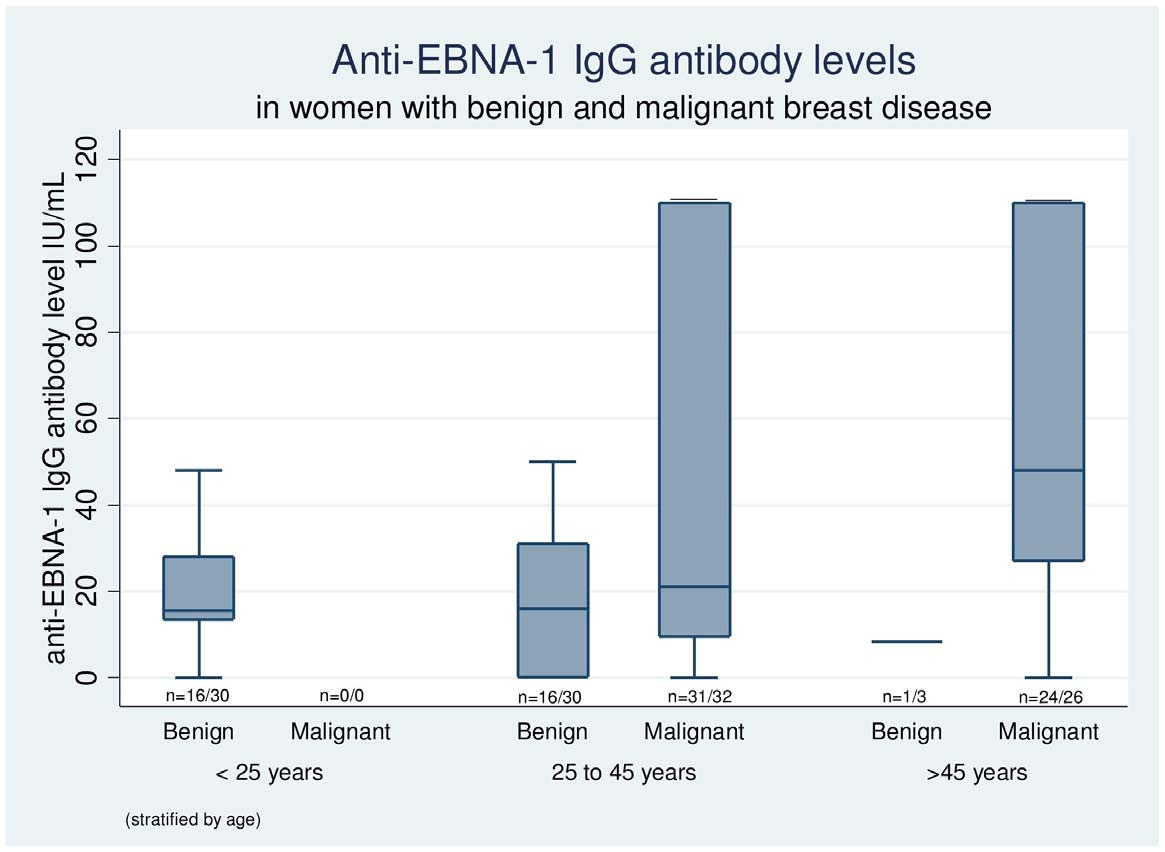
Box plot showing anti-EBNA-1 IgG antibody levels in women with benign and malignant breast disease, stratified by age. The central horizontal line in the box indicates the median value, edges of the box indicate inter-quartile range, and the whiskers indicate values at 5^th^ and 95^th^ percentiles. The values at the bottom of the box indicate the number of women in whom serology was done, out of the total number of eligible women in particular category. The mean IgG levels in 25 to 45 year age group were significantly higher in women with malignant breast disease (48.15; SD 47.38 IU/ml) as compared to those with benign breast disease (17.37; SD 16.89 IU/mL) (p = 0.01).

Serology for EBNA-1 was done in 55/58 (94.8%) cases and 33/63 (54.3%) controls. Using manufacturer determined cut-off of 3 IU/ml, 50/55 (90.9%) cases and 27/33 (81.8%) controls were seropositive for anti-EBNA-1 IgG. Mean anti-EBNA-1 IgG levels were significantly higher for cases as compared to controls, 54.22 IU/ml (SD 46.70) (Median 34, range 0 to 110) versus 18.68 IU/ml (SD 14.80) (Median 16, range 0 to 50). We also analyzed serology results stratified by age. None of the women aged less than 25 years had malignant breast disease. Only three women above the age of 45 years had a benign breast disease. In the age-group of 25 to 45 years, 30 women had benign and 32 had malignant breast disease. The 16 of the 30 women in this age group who had a benign breast disease and in whom serology was performed, mean IgG levels (17.37 IU/mL; SD 16.89) were significantly lower as compared to those with malignant breast disease (48.15; SD 47.38 IU/ml) (p = 0.01) (figure 2).

**Figure 3:**
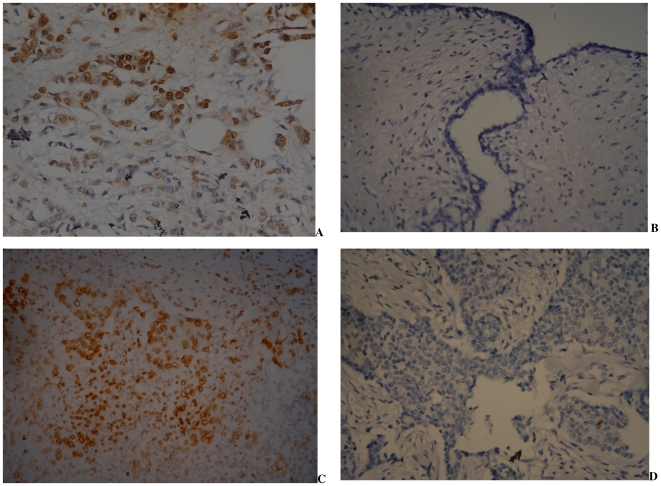
Immunohistochemical detection of EBNA-1 in breast tissue specimens. A. Tumor cells (infiltrating ductal carcinoma) showing intense granular nuclear positivity for EBNA-1 antibody. B. Benign breast tissue negative for EBNA-1; C. Positive control (nasopharyngeal carcinoma tumor cells showing nuclear positivity for EBNA-1) slide; D. Negative control (no signal is seen in infiltrating ductal carcinoma cells without application of primary antibody). All photographs and in 400x original magnification.

Of the 51 cases in whom IHC for EBNA-1 was performed, 28 were positive (54.9%). In the positive slides, about 3 to 50% of the tumor cells showed intense nuclear granularity. No positivity was noted in the infiltrating lymphocytes, surrounding stroma or adjacent non-cancerous tissue (figure 3). All tested 30 controls were negative for EBNA-1 expression by IHC. Of 48 cases in whom both serology and IHC were performed, 25 (52.1%) were concordant positive, 2 (4.6%) concordant negative, while remaining 21 (43.5%) were positive by one of the two tests. Among 30 controls where both tests were done, none was concordant positive, 6 (20%) were concordant negative, while 24 (80%) were positive by serology alone. Among 51 cases in whom IHC was done, EBNA-1 positivity was not found to be associated with age, clinical or reproductive characteristics or tumor aggressiveness. (Table 2)

**Table 2:** Demographic, reproductive and tumor severity amongst cases (n = 51) with positive or negative IHC.

	IHC EBNA-1 positiveN = 28 (55%)	IHC EBNA-1 negativeN = 23 (46%)	P value
Age (years)	45.5 (8.7)	47.0 (10.4)	0.62
Duration (months)	7.3 (9.3)	7.6 (9.5)	0.91
Age of menarche (years)	14 (1.4)	13.7 (0.76)	0.32
Pregnancies (no)*	2.82 (1.49)	3.04 (1.82)	0.63
Number of children*	2.64 (1.47)	2.73 (1.28)	0.80
Never lactated*	3 (10.7%)	2 (8.7%)	0.81
Tumor grade *			
IIIIII	1 (3.6%)12 (42.8%)15 (53.5%)	1 (4.5%)8 (36.3%)13 (59.1%)	0.89
Tumor size*			
T1T2T3T4	0 (0%)11 (39.3%)13 (46.4%)4 (14.3%)	2 (8.7%)8 (34.7%)10 (43.5%)3 (13.0%)	0.46
Lymph-node involvement*			
N0N1N2N3	8 (28.6%)7 (25.0%)8 (28.6%)5 (17.9%)	8 (34.7%)8 (34.7%)5 (21.7%)2 (8.7%)	0.66
Distant metastasis*			
M0M1	26 (92.8%)2 (7.1%)	22 (95.6%)1 (4.3%)	0.67

All values indicate mean (standard deviation) unless stated otherwise.

* Number (percentage).

## Discussion

In the current study we found that about 55% of breast cancer cases showed EBNA-1 expression in tumor cells by IHC, while all the controls with benign breast disease were negative. On the other hand although both cases as well as controls had a high sero-prevalence of EBV infection (proportion positive for EBNA-1 IgG antibodies 90.90% and 81.8% respectively), but IgG levels were significantly higher in cases as compared to controls. These findings suggest that EBV infection may have a role in causing breast cancer.

Our results support the findings of some studies where expression of EBNA-1 has been noted in cases of breast cancer. (Table 3) Few of these studies also showed presence of EBV DNA on polymerase chain reaction (PCR).[10], [12], [13] Like other studies we also observed focal positivity in breast cancer cases. No positivity in cases of benign breast diseases has also been reported by Preciado etal [12], Roberio-Silva etal [14] and Fawzy etal. [13]. Grinstein etal [15] and Roberio-Silva etal [14] have observed nuclear positivity for EBNA-1 in atypical breast hyperplasias and carcinoma in situ. Some investigators have reported that EBV expression is more commonly seen in tumors of higher grade,[10], [16] no such association between tumor grade and EBNA-1 expression was noted in our study. Only two patients with malignant breast disease in our study had a low grade malignancy (tumor grade I), and all remaining had high tumor grades (II, and III). Thus a lack of contrast in disease severity in our study population is a likely explanation for why our results were different from previous reports.

**Table 3:** Previous published studies which evaluated EBNA-1 by IHC on breast cancer specimens.

First author, year	EBNA-1 positive/Total	% of cells stained
Bonnet[10] 1999	9/9*	5–30
Brink[27] 2000	1/5*	NA
Chu[31]2001	12/48	<1
Grinstein[15] 2002	14/33	5–30
Deshpande [32]2002	0/43	0
Hermann [7]2003	0/59	0
Roberio-Silva[14] 2004	29/73	NA
Preciado [12] 2005	24/69	5–60
Shereen Fawzy[13] 2008	10/40	5–60

* IHC was done on EBV-DNA PCR positive samples only.

Only two previous studies have looked for serological evidence of infection in breast cancer patients. [17], [18]. Traditional marker of EBV infection was investigated in only one study and the authors measured EBV IgG levels in stored plasma of 208 breast cancer patients and169 controls. Similar seroprevalence of EBV infection was noted in cases and controls (97 and 96 percent respectively), but in contrast to the present study, mean IgG levels were not found to be higher in cases as compared to controls (2.65 vs 2.57).[18]

The mean age of cases and controls (47.22 and 27.52 years respectively) in our study is significantly different, hence the serological findings need to be interpreted with caution. It could be argued that since cases belong to an older age group, increased antibody levels against EBNA-1 are simply because of multiple re-infections which occur over a period of time. However, our age stratified analysis shows that even in 25 to 45 years of age category, the IgG levels in women with malignant breast disease were significantly higher. Yasui et al [19] hypothesized that delayed primary EBV infection is associated with a heightened immune response and an elevated risk of breast cancer. Though in this study we cannot ascertain the timing of infection in cases, but in comparison to controls they definitely had a heightened immune response against EBNA-1.

There were 18 cases which were negative for EBNA-1 expression on IHC but had positive serology results and 3 cases which were positive for EBNA-1 on IHC but negative on serology. The reason for results of these latter three cases is not exactly clear. The most likely reason could be technical. We have used stored sera and hemolysis, lipemia or contaminated sera could result into false positive or false negative results. It is also known that most but not all individuals form anti-EBNA-1 antibodies after EBV infection and in some cases these antibodies disappear secondarily and thus do not persist lifelong. Such cases should be investigated for presence of viral capsid antigen antibodies before labelling them as seronegative.[20]

Many investigators have questioned the role of EBV as a primary etiologic agent for breast cancer. Major reasons for this controversy have been firstly, the inconsistency of results in different studies and secondly, detection of EBV expression in only a subset of tumor cells. Initial studies focused primarily on association of medullary carcinoma with EBV infection, and yielded negative results.[21], [22]. Subsequently, Laberque et al [23] raised the possibility of association of invasive ductal and lobular carcinoma with EBV infection, but the results from various follow-up studies have yielded variable results.[7], [10], [12], [13], [14], [15], [16], [24], [25], [26], [27], [28], [29], [30], [31], [32], [33], [34], [35], [36], [37], [38], [39], [40], [41], [42], [43] Part of this variability can be explained by differences in techniques and antigen targets. Broadly three techniques have been used to determine EBV expression in breast cancer cells IHC (EBNA-1, EBNA-2, LMP), In-situ hybridization (ISH) for EBV encoded nuclear RNA (EBER), and PCR for EBV-DNA. IHC for EBNA-1 has yielded higher positivity, however concerns remain regarding the specificity of the antibody used in some of these studies.[34], [44] The studies which have used EBNA-1 as a target antigen are summarized in table 3. Studies which have used LMP-1 and EBNA-2 have mostly yielded negative results. This can be explained by the fact that these antigens are differentially expressed in EBV associated malignancies.[5] ISH targeting EBER is considered as a gold standard for detecting latent EBV infection. However the results of the studies detecting EBER by ISH in breast cancer specimens have been mostly negative. This discrepancy may be due to non-expression of EBER in breast cancer.[5] Conventional PCR based studies have been unable to differentiate between EBV-DNA of tumor cells from EBV-DNA of circulating lymphocytes. Use of Laser micro-dissection to separate out tumor cells alone, followed by PCR testing can potentially solve this limitation, yet the results based on these studies have been contradictory. [16], [28], [33], [34]

Secondly, the reason for only some of the tumor cells being positive for EBV expression could be because of low expression of protein in tumor cells or because of poor accessibility of the antibody to the antigenic site. Even in NPC, which is known to be caused by EBV infection, not all tumor cells are stained by the antibody.[10] Another possible reason of variable IHC staining could be that EBV is a hit and run virus, and after transformation of breast epithelial cells, it gets lost from tumor cells [5], [10] Arbach et al [40] demonstrated that EBV genome is heterogeneously distributed amongst morphologically indistinguishable tumor cells which could account for the variable expression of EBNA-1 in tumor cells. They also suggested that infection with EBV at a late stage of tumor development may enhance it's oncogenic properties like invasion, angiogenesis and metastasis. They demonstrated that in vitro infection of breast carcinoma cells by EBV confers paclitaxel resistance and evokes overexpression of a multi drug resistance gene so even if a small population of breast carcinoma cells are infected by EBV, the impact of EBV infection on efficacy of anticancer treatment would be of importance.[40]

This study has several strengths. Firstly, this is the first study looking for evidence of association of EBV infection with breast cancer which has been conducted in India. In India breast cancer occurs at a younger age, and traditional reproductive risk factors are uncommon. In this setting looking for novel etiological factors becomes important. Secondly, we looked for evidence of EBV infection in both serum as well as breast tissue specimens in prospectively collected samples. Thirdly, we employed an in-situ technique to demonstrate viral protein in breast tissue. Lastly, we demonstrated expression of EBNA1, a protein essential for maintaining viral episome. Presence of this protein helps to establish EBV infection in tumor cells.

This study also has certain limitations. Firstly our sample size is small and the results cannot be generalized to normal population. Secondly we have not been able to find age matched controls for each case as in general the mean age of controls is lower than that of cases. This is a major limitation in statistical comparison of antibody levels between the two groups. Thirdly we have looked for expression of only one viral protein: EBNA-1, ideally we should have investigated for multiple proteins present in different forms of viral latency seen in other EBV associated tumors. Also we have investigated for presence of EBNA-1 expression in tissues only by IHC and that too by using only one monoclonal antibody. In-order to prove specificity of the antibody we have used, we need to reproduce our results by using another monoclonal antibody against EBNA-1.

Molecular evidence of association is also required to substantiate our results.

Our results show that expression of EBNA-1 is seen in a large subset of breast cancer cases and a high immunological response to EBNA-1 is also seen in these patients. Considering the magnitude of breast cancer, role of EBV in either causation or progression of breast cancer even in a small subset of breast cancer would be of paramount importance. Further research is warranted to define the exact role of EBV in the etiology or progression of breast cancer as it would help in designing preventive, early detection and treatment strategies.
